# BIRC5 Modulates PD-L1 Expression and Immune Infiltration in Lung Adenocarcinoma

**DOI:** 10.7150/jca.69236

**Published:** 2022-08-21

**Authors:** Teng Ma, Jie Gu, Haoyu Wen, Fengkai Xu, Di Ge

**Affiliations:** Department of Thoracic Surgery, Zhongshan Hospital, Fudan University, Shanghai, China.

**Keywords:** LUAD, BIRC5, PD-L1, Immune infiltration

## Abstract

**Background:** Lung adenocarcinoma (LUAD) is the most prevalent thoracic cancer with the highest incidence and mortality worldwide. Baculoviral IAP Repeat Containing 5 (BIRC5) is well studied in many malignancies, its prognosis value and correlation with the tumor microenvironment (TME) in LAUD remains largely elusive.

**Methods:** The Wilcoxon signed-rank test and logistic regression were used to evaluate the relationship between clinical features and BIRC5 expression in LUAD. To assess the impact of BIRC5 on prognosis, the Kaplan-Meier plotter analysis and Cox regression were used, as well as a receiver operating characteristic (ROC) curve and nomogram. Gene set enrichment analysis (GSEA) and single-sample gene set enrichment analysis (ssGSEA) were recruited to predict the association between BIRC5 and immune cell infiltrations. Furthermore, qRT-PCR and western bolt were utilized to confirm gene expression on mRNA and protein levels. The proliferation of A549 and H1299 cells was evaluated using CCK8 and EdU assay. Cell mobility was tested by transwell assay and wound healing assay. Detection of PD-L1 and infiltrated CD8 T cells in xenograft tumors was done by flow cytometry.

**Results:** BIRC5 expression was found to be substantially greater in LUAD patients. According to KM-plotter analysis, patients with high levels of BIRC5 had shorter survival rates. Multivariate Cox analysis revealed that elevated BIRC5 expression was an independent risk factor for OS and PFS in LUAD patients. High BIRC5 expression was predicted to be associated with chemokine activity and immune cell chemotaxis, whereas ssGSEA suggested that BIRC5 is highly associated with CD8 T cell infiltration and PD-L1 levels. *In vitro* experiments suggested overexpression of BIRC5 promoted the proliferation, mobility, and PD-L1 level of A549 cells, and vice versa in H1299 cells. Furthermore, *in vivo* study suggested elevated tumor weight and PD-L1 levels in xenograft tumors generated from LLC cells with overexpressed BIRC5.

**Conclusion:** BIRC5 promotes lung adenocarcinoma progression by modulating PD-L1 expression and inducing tumor immune evasion.

## Background

Lung adenocarcinoma (LUAD) is the most prevalent kind of thoracic cancer with the highest incidence and mortality worldwide 1. The wide invasion and metastasis of LUAD contributes to the unsatisfying survival of LUAD patients 2. Immune-related processes and indicators have a role in the carcinogenesis and development of LUAD, and immune-related targeted therapy offers a viable therapeutic option for LUAD 3-5. According to a recent study on the advanced stage of non-small cell lung cancer (NSCLC), the 5-year overall survival (OS) rate in the immunotherapy group was approximately double that in the standard chemotherapy group (31.9% vs. 16.3%) 6. However, patients with programmed death ligand-1 (PD-L1) tumor proportion score (TPS) < 50% had been rarely included in those clinical trials due to poor expected response. Thus, there has always been an urgent need to discover novel immune checkpoints or indicators for the diagnosis and therapy in LUAD.

Baculoviral IAP Repeat Containing 5 (BIRC5, also named Survivin) is defined as a member of the inhibitor-of-apoptosis proteins (IAPs) family, which has dual biological activities that regulate both mitosis and apoptosis in embryogenesis of embryonic cells and carcinogenesis of malignant diseases 7-9. Aberrant expression of BIRC5 in various malignancies has been confirmed closely associated with cell cycle, proliferation, migration, immune infiltration, and the communication between tumor cells and the tumor microenvironment (TME) 10, 11. Admitted that BIRC5 has been well studied in kinds of malignancies, its prognosis value and correlation with TME remains largely elusive in LAUD.

In this study, we firstly focused on BIRC5 expression and its predictive significance in LUAD patients. The underlying mechanisms were next investigated in The Cancer Genome Atlas (TCGA) LUAD data set. The association between BIRC5 levels and tumor-infiltrating cells levels was estimated. Finally, we performed *in vitro* and *in vivo* experiments that demonstrated both tumor-promoting and immunosuppressing roles of BIRC5 in LUAD.

## Materials and methods

### Data Source and patients recruitment

The transcriptome RNA-sequencing data and the clinical information of 535 LUAD samples were obtained from the TCGA (https://tcga-data.nci.nih.gov/tcga/) 12. The inclusion criteria: patients diagnosed with primary LUAD; patients whose tumor samples have been tested with transcriptome RNA-secquencing; patients whose overall survival information was documented; patients who received no previous treatment. The exclusion criteria: patients who had other malignancy histories; patients whose overall survival information was not available; repeated samples from the same patients. A total of 513 LUAD samples out of 535 from TCGA met our criteria and were recruited for this study.

### Gene Set Enrichment Analysis (GSEA)

Using the “cluster Profiler” R program, we recruited GSEA to explore possible biological processes and pathways 13, 14. The Molecular Signatures Database (MSigDB) was used to obtain the c5.all.v7.2.symbols.gmt (Gene ontology) and c2.cp.v7.2.symbols.gmt (KEGG). Gene sets were considered statistically significant with |NES|>1, adj.p<0.05, and FDR<0.05.

### Immune Cells Infiltration of ssGSEA

Single-sample gene set enrichment analysis (ssGSEA) from the R package “Gene Set Variation Analysis” (GSVA) 15 was used to analyze LUAD immune infiltration. The immune cells infiltration levels were measured using gene expression profiles 16. Spearman correlation was used to investigate the relationship between BIRC5 levels and LUAD immune cell infiltrations.

### Cell Cultures

The Chinese Academy of Sciences provided human lung epithelial cell BEAS-2B and NSCLC cell lines A549, H1650, H1975, PC9, SPC-A1, and H1299. All cells were cultured in RPMI-1640 medium (Gibco, USA) with 10% fetal bovine serum (FBS, Gibco, USA) and antibiotics (100 units/mL penicillin and 100 ug/mL streptomycin) in a humidified incubator with 5% CO_2_ at 37 °C.

### Quantitative Real-Time Polymerase Chain Reactions (qRT-PCR) of Cell Lines

Total RNA extraction and reverse transcription were carried out in the same manner as in prior work 17. The mRNA levels were calculated based on the 2^-ΔΔCt^ method, and GAPDH was used as an internal reference. The qRT-PCR primers used in this study were as follows:

BIRC5 forward primer, 5ʹ-CCACTGAGAACGAGCCAGACTT-3ʹ; BIRC5 reverse primer, 3ʹ-GTATTACAGGCGTAAGCCACCG-5ʹ; GAPDH forward primer, 5ʹ-GTCTCCTCTGACTTCAACAGCG-3ʹ; GAPDH reverse primer, 3ʹ- ACCACCCTGTTGCTGTAGCCAA-5ʹ; PD-L1 forward primer, 5ʹ-TGCCGACTACAAGCGAATTACTG-3ʹ; PD-L1 reverse primer, 5ʹ-CTGCTTGTCCAGATGACTTCGG-3ʹ; LAG3 forward primer, 5ʹ-GCAGTGTACTTCACAGAGCTGTC-3ʹ; LAG3 reverse primer, 5ʹ-AAGCCAAAGGCTCCAGTCACCA-3ʹ.

### CCK8 and EdU assay

The CCK8 assay was performed using a CCK8 Cell Proliferation Kit (Beyotime, China) according to the manufacturer's instructions. After adding CCK8 reagents, the absorbance at 450 nm was measured. EdU staining was carried out using the EdU kit (RiboBio, China) according to the manufacturer's instructions. EdU-positive rate = EdU-positive cell count/cell count *100%.

### Transwell migration and wound healing assay

Wounds were made with pipette tips on A549 or H1299 cell monolayers, and the cells were cultivated for 48 hours at 37 °C with 5% CO_2_. Sections of the wounds were imaged before and after incubation. The degree of wound repair was calculated using ImageJ (NIH, USA). Migration assays were performed using a transwell with a pore size of 8.0 um (Millipore), according to the manufacturer's instructions.

### Western blot analysis

Western blot analyses were carried out following the previously described standard protocols 18. Anti-Survivin, anti-PD-L1, anti-β-actin, anti-phospho-mTOR (Ser2448), and anti-mTOR were purchased from Cell Signaling Technology (CST).

### Tumor xenograft assay

The xenograft tumors were generated in 6-week-old C57BL/6 mice. One million LLC cells pre-transfected with Lv-BIRC5 or Lv-NC were injected subcutaneously into the left flank of mice. Three weeks after the injection, tumor tissues were harvested. Complied with the declaration of Helsinki, this study has been approved by the ethics committee of Zhongshan Hospital, Fudan University, Shanghai, China.

### Flow cytometry analysis

Tumors were collected three weeks after injection and digested in RPMI for 40 minutes with Collagenase (Sigma, USA) and DNase I (Roche, USA). Following that, the single-cell suspension was filtered through a 100-um micron filter and stained with antibodies for flow cytometry analysis. The FlowJo software (V10) was used for data analysis.

### Statistical Analysis

Box plots were used to compare BIRC5 expression in LUAD patients to normal ones from GTEx samples.

The median BIRC5 level was used as the cut-off value. The relationship between clinical characteristics and BIRC5 expression in LUAD was next investigated using the Wilcoxon signed-rank test and logistic regression. We evaluated the overall survival (OS), first progression (FP), and post-progression survival (PPS) in two groups with high and low BIRC5 levels, using The Kaplan-Meier plotter (http://kmplot.com/analysis/). The diagnostic value of BIRC5 was determined using a receiver operating characteristic (ROC) curve, and the area under the ROC curve serves as the diagnostic value.

To screen possible prognostic variables, univariate and multivariate Cox analyses of the TCGA-LUAD dataset were undertaken. Following that, a multivariate Cox analysis was performed to confirm the independent prognostic variables of BIRC5 expression, and a nomogram was created to predict the OS of LUAD patients for 3-time frames. We used GraphPad Prism 8.0 for data analysis. Statistical data were expressed as mean ± SD values. Statistical significance was determined with a t-test. The correlation analysis was assessed with Pearson's correlation. P < 0.05 was considered statistically significant.

## Results

### BIRC5 is highly expressed in LUAD

**Figure 1:**
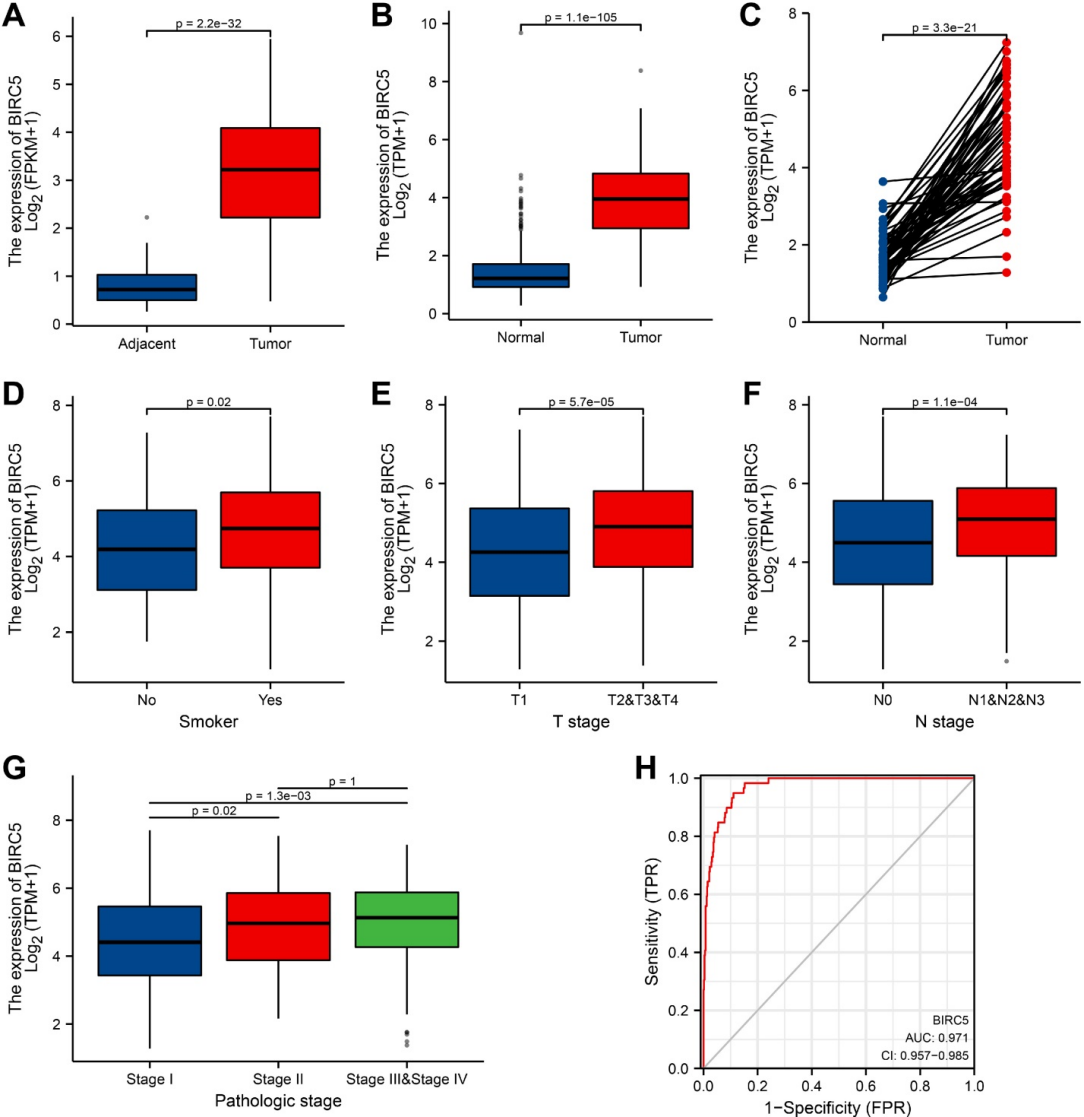
** High BIRC5 expression is correlated with clinicopathologic features in LUAD patients from the TCGA-LUAD cohort. (A)** Wilcoxon signed rank-sum test was applied to analyze the levels of BIRC5 in LUAD tissues and adjacent LUAD tissues. **(B)** Wilcoxon signed rank-sum test was applied to detect the levels of BIRC5 in normal samples of GTEx combined adjacent LUAD tissues and LUAD tissues. **(C)** The expression levels of BIRC5 in 59 LUAD samples and matched adjacent samples. **(D)** Box plot assessing BIRC5 expression of patients with LUAD according to smoking history. **(E)** Box plot assessing BIRC5 levels of LUAD patients according to T stage. **(F)** Box plot assessing BIRC5 levels of LUAD patients according to N stage. **(G)** Box plot assessing BIRC5 levels of LUAD patients according to pathologic stage.** (H)** ROC curve for BIRC5 expression levels in normal samples of GTEx combined adjacent LUAD tissues and LUAD samples.

We examined the expression of BIRC5 and discovered that BIRC5 expression was significantly higher in LUAD tissues than in adjacent tissues (p = 2.2e-32) (Figure 1A). Simultaneously, the expression levels of BIRC5 in normal GTEx merged adjacent tissues and LUAD samples were analyzed, and it was discovered that BIRC5 was dramatically up-regulated in LUAD (p = 1.1e-105) (Figure 1B). Furthermore, BIRC5 expression was elevated in tumor tissues across 59 LUAD samples and matched normal samples (p = 3.3e-21) (Figure 1C). The receiver operating characteristic (ROC) curve was used to assess the efficacy of BIRC5 expression in GTEx coupled adjacent tissues and LUAD samples. The area under the curve (AUC) was 0.971, indicating an extremely high diagnostic value of BIRC5 in LUAD (Figure 1H).

### Correlation between BIRC5 expression and clinical characteristics

Clinical characteristics of 513 recruited LUAD patients were gathered from the TCGA database. Patients were classified into high- and low-BIRC5 level groups based on the mean value of BIRC5 expression, and we then used the logistic regression and Wilcoxon signed-rank test to examine the relationship between BIRC5 levels and clinical features (Table 1). Higher BIRC5 level was correlated with smoking (p = 0.02) (Figure 1D), T classification (p = 5.7e-05) (Figure 1E), lymph node metastases (P = 1.1e-04) (Figure 1F), and progressed TNM stage (Figure 1G). The results of univariate analysis using logistic regression demonstrated that BIRC5 expression was associated with poor prognostic clinical characteristics in patients with LUAD (Table 2). High BIRC5 expression was significantly correlated with gender (OR = 1.961, 95% CI = 1.381-2.795, p <0.001), smoking (OR=1.681, 95% CI = 1.021-2.807, p = 0.043), T classification (T2&T3&T4 vs. T1: OR = 2.055, 95% CI = 1.413-3.008, p <0.001), N stage (N1&N2&N3 vs. N0: OR = 1.886, 95% CI = 1.298-2.754, p <0.001 ) and primary outcome (PD&SD vs. PR&CR: OR = 1.600, 95% CI = 1.028-2.509, p = 0.039).

### The value of BIRC5 expression in predicting LUAD survival

**Figure 2:**
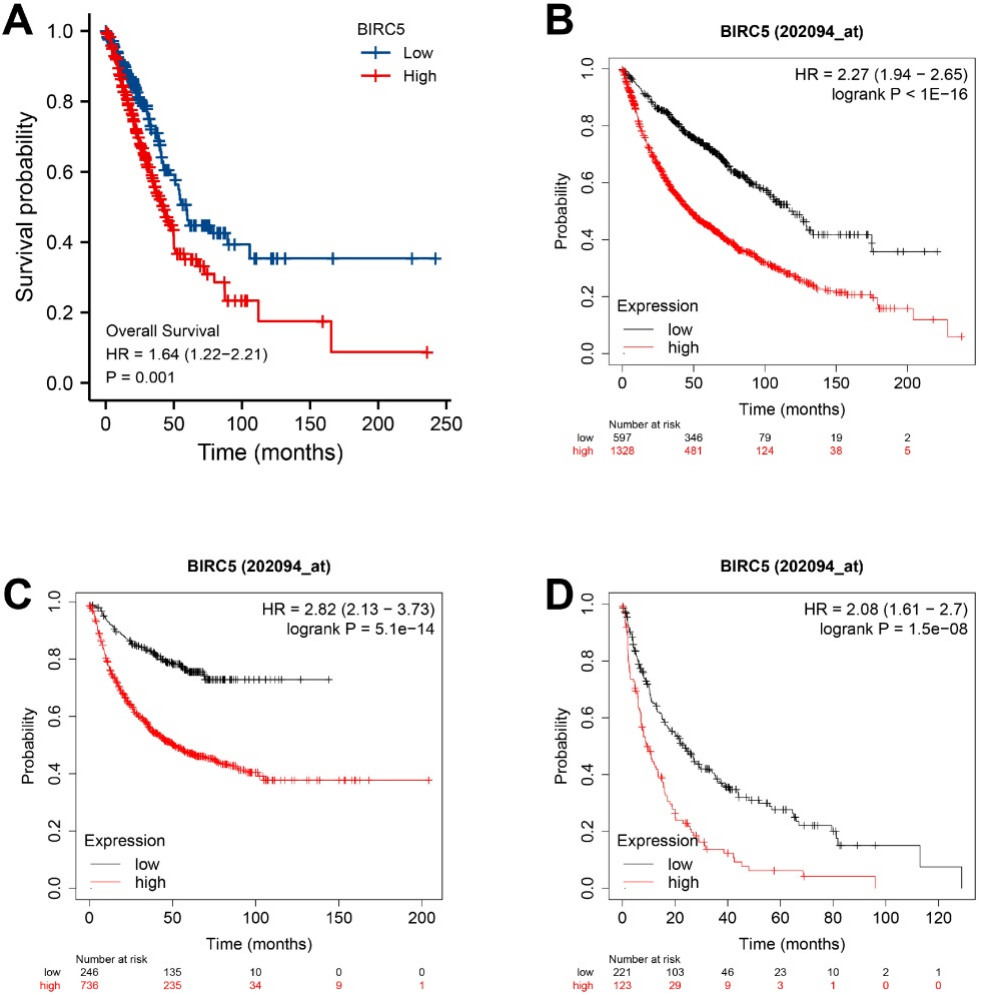
** The independent risk value of BIRC5 expression levels in LUAD. (A)** The survival analysis of overall survival (OS) of the TCGA-LUAD cohort. **(B)** Kaplan-Meier survival analysis of OS. **(C)** Free progression (FP). **(D)** Post-progression survival (PPS).

The TCGA-LUAD dataset survival analysis revealed that high BIRC5 level was correlated with poor OS (p = 0.001) (Figure 2A), and the Kaplan-Meier plotter analysis indicated its association with poor OS (HR=2.27, 95% CI=1.94-2.65, p = 1.0e-16) (Figure 2B); free progression (FP, HR=2.82, 95% CI=2.13-3.73, p = 5.1e-14) (Figure 2C); and post-progression survival (PPS, HR=2.08, 95% CI=1.61-2.7, p = 1.5e-08) (Figure 2D). Univariate and Multivariate Cox analysis revealed a significant correlation between high BIRC5 with poor OS (Supplementary Figure 1A) and PFS (Supplementary Figure 1B) in LUAD. The nomogram predicts the 1, 3, 5-year OS in the TCGA-LUAD cohort using age, T, M, N classification, pathologic stage, residual tumor, and BIRC5 expression (Supplementary Figure 1C).

### BIRC5 involved gene-sets in GSEA

**Figure 3:**
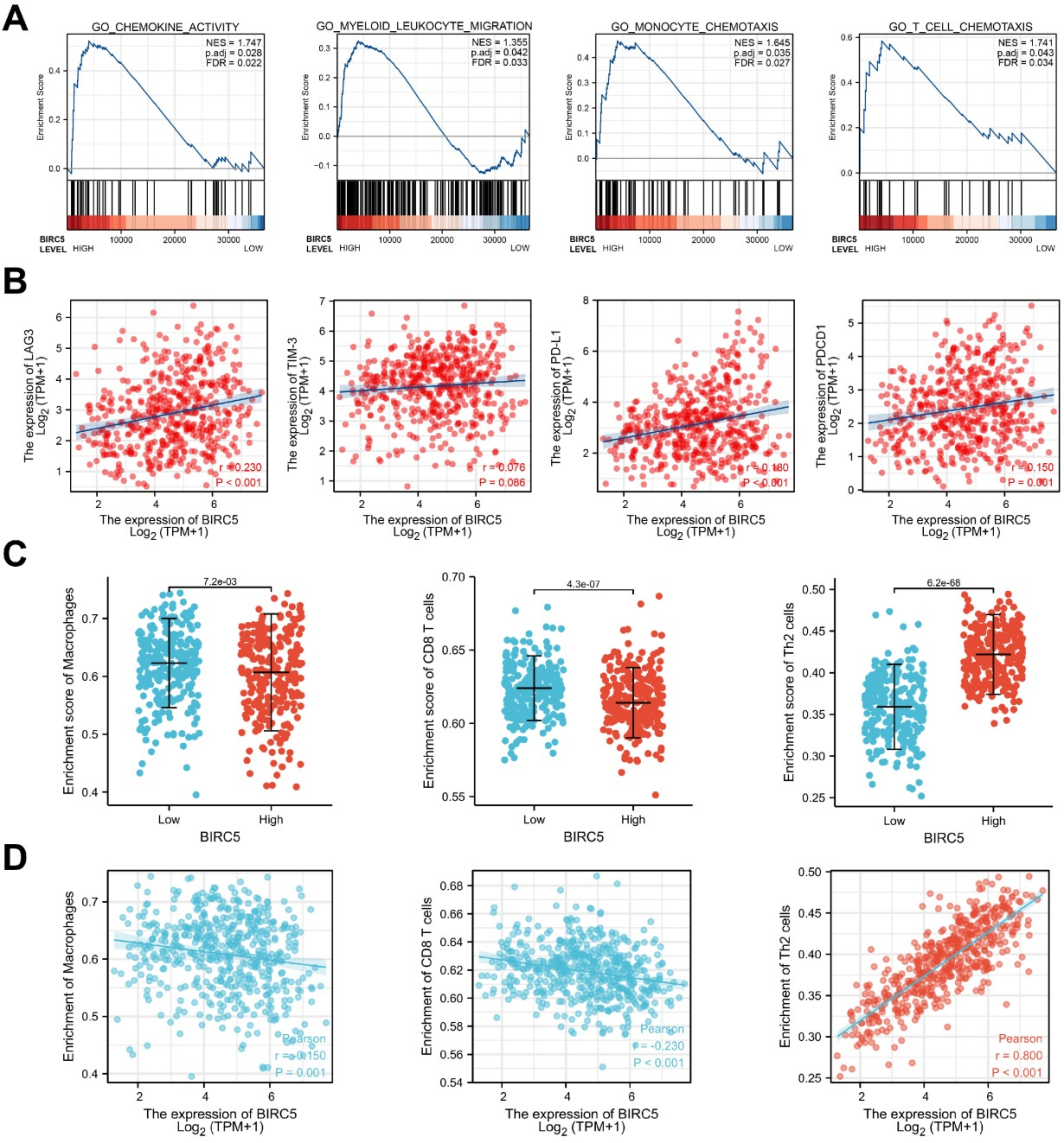
** Immune Cell Infiltration Analysis of BIRC5 in the TCGA-LUAD. (A)** Gene set enrichment analysis (GSEA) based on BIRC5 expression in the TCGA-LUAD cohort. **(B)** Pearson analysis between BIRC5 expression and levels of LAG3, TIM-3, PD-L1, and PDCD1. **(C)** The correlation between BIRC5 expression and enrichment of immune cells in TCGA-LUAD cohort. **(D)** Enrichment scores of BIRC5 levels in Macrophages, CD8 T cells, and Th2 cells in the TCGA-LUAD cohort. **(E)** The correlation between BIRC5 expression and enrichment of Macrophages, CD8 T cells, and Th2 cells in TCGA-LUAD cohort.

Gene set enrichment analysis (GSEA) was used to investigate BIRC5-involved pathways. We discovered that chemokine activity and several immune cell chemotaxis pathways were enriched in the high BIRC5 group, as shown in Figure 3A.

### Immune cell infiltration analysis depends on the BIRC5 level

By Pearson analysis of the TCGA-LUAD cohort, levels of hub genes regulating T-cell exhaustion (LAG3, TIM-3, PD-L1, and PDCD1) were found positively correlated with the expression of BIRC5 (Figure 3B).

Next, we investigated the relationship between BIRC5 levels and the degree of immune cell infiltration via single-sample gene set enrichment analysis (ssGSEA). The results revealed that BIRC5 expression was associated with the infiltration of various kinds of immune cells (Figure 3C). Macrophages, CD8 T cells, and Th2 cells, the most crucial tumor-associated immune cells, were predicted significantly correlated with BIRC5 levels in the TCGA-LUAD cohort (Figure 3D and E). These results indicate BIRC5 could play a significant role in immune infiltration and exhaustion in LUAD.

### Validation of BIRC5 expression in cell lines

**Figure 4:**
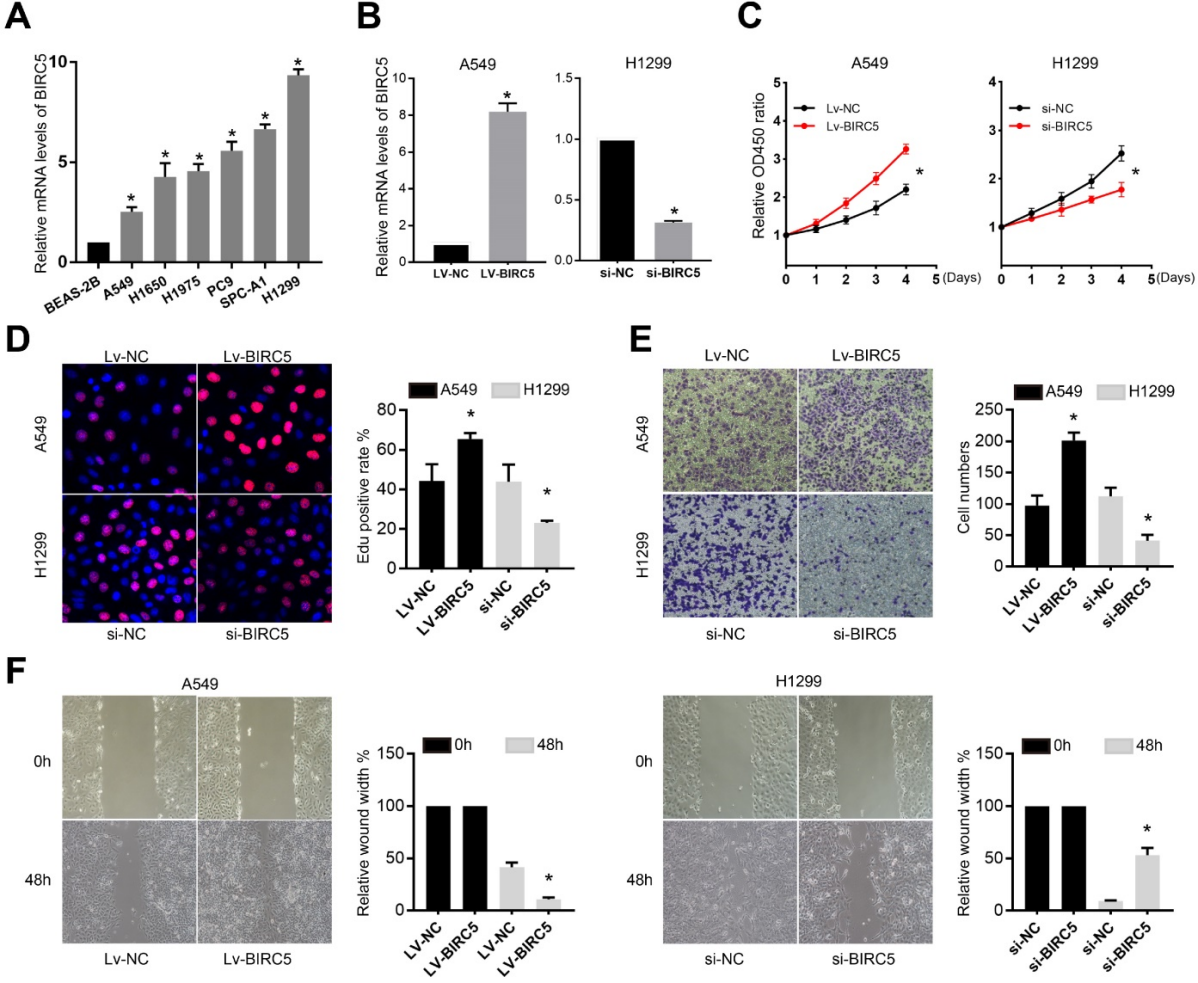
** BIRC5 was upregulated in lung cancer cell lines and promoted cell proliferation and mobility *in vitro*. (A)** RT-qPCR analysis of BIRC5 levels in the BEAS-2B cell line and several lung cancer cell lines. **(B)** Relative BIRC5 expression in A549 and H1299 cells after transfection Lv-BIRC5/si-BIRC5 and respective negative controls. **(C)** CCK8 analysis was recruited to test A549 or H1299 cells proliferation after transfection of Lv-BIRC5 or si-BIRC5, respectively**. (D)** Cell proliferation was tested by EdU staining and calculated as EdU+ cells/total cells. **(E)** Transwell assays were performed to test the migration of A549 or H1299 cells after transfection of Lv-BIRC5 or si-BIRC5, respectively. **(F)** Wound-healing assays indicated ectopic expression of BIRC5 promoted A549 cell migration and knockdown of BIRC5 suppressed H1299 cell migration, *p<0.05.

The qRT-PCR analysis was performed in several NSCLC cell lines (A549, H1650, H1975, PC9, SPC-A1, and H1299) and one normal lung epithelial cell line BEAS-2B. Levels of BIRC5 were found significantly higher in malignant cells (Figure 4A). A549 cells were infected with a BIRC5-overexpressed lentivirus (Lv-BIRC5) or negative control (Lv-NC). H1299 cells were infected with a small interfering RNA targeting BIRC5 (si-BIRC5) or a negative control si-RNA (si-NC) (Figure 4B).

### BIRC5 promotes the proliferation and migration of NSCLC cell lines *in vitro*

Using the Cell Counting Kit-8 (CCK-8) and 5-ethynyl-2'-deoxyuridine (EdU) proliferation assays, we discovered that knocking down of BIRC5 greatly decreased the proliferation of H1299 cells, whereas A549 cell proliferation was enhanced by BIRC5 ectopic expression (Figure 4C and D). The transwell assay (Figure 4E) and the wound healing assay (Figure 4F) revealed that si-BIRC5 treatment significantly reduced the migration capacity of H1299 cells, whereas BIRC5 overexpression increased it in A549 cells. These *in vitro* studies suggested that BIRC5 promotes NSCLC cell proliferation and migration.

### BIRC5 promotes PD-L1 expression via mTOR activation

**Figure 5:**
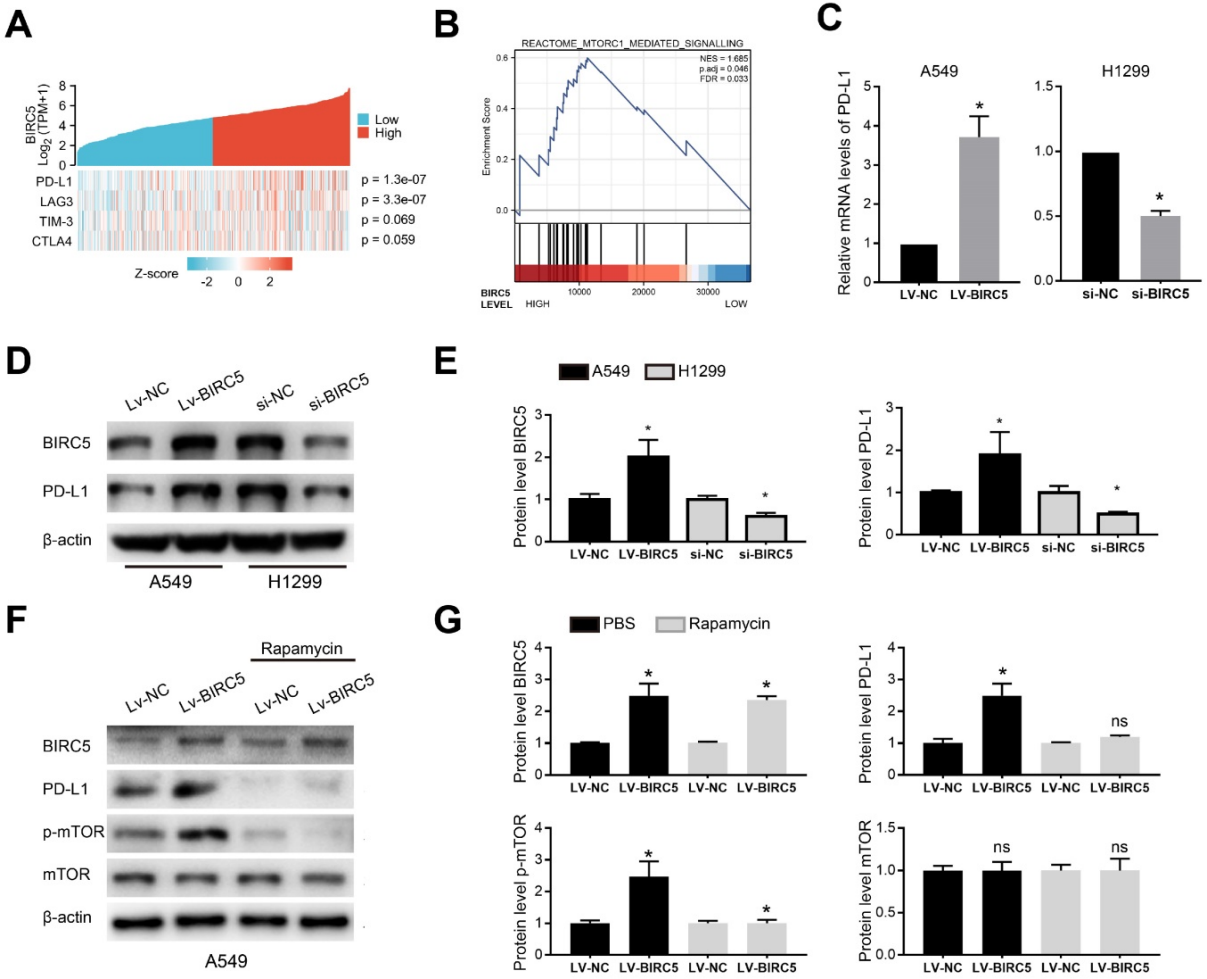
** BIRC5 modulates PD-L1 expression via mTOR activation. (A)** A single gene co-expression heat map using data from the TCGA-LUAD cohort showed BIRC5 expression was positively correlated with PD-L1 levels (p=1.3e-07) and LAG3 levels (p=3.3e-07). **(B)** The mTORC1 mediated signaling was enriched via GSEA based on BIRC5 expression in the TCGA-LUAD cohort. **(C)** PD-L1 mRNA levels were higher in A549 cells transfected with Lv-BIRC5 whereas lower in H1299 cells transfected with si-BIRC5, compared to its negative controls. **(D and E)** PD-L1 protein levels were higher in A549 cells transfected with Lv-BIRC5 whereas lower in H1299 cells transfected with si-BIRC5, compared to the negative controls. Predicted protein molecular weight is annotated.** (F and G)** Rapamycin partially reversed the PD-L1 overexpression caused by Lv-BIRC5 on the protein level, *p<0.05.

A heat map of four T-cell exhaustion-related genes expression in the LUAD cohort from the TCGA database indicated that BIRC5 expression was significantly correlated with PD-L1 (p=1.3e-07) and LAG3 (p=3.3e-07) (Figure 5A). GSEA of the TCGA-LUAD cohort indicated the BIRC5 level was positively correlated with the activation of mTORC1 mediated signaling (NES=1.685, FDR=0.033) (Figure 5B). The qRT-PCR analysis showed BIRC5 overexpression significantly increased the mRNA level of PD-L1 in A549 cells, whereas si-BIRC5 treatment decreased it in H1299 cells (Figure 5C). Similar results were determined on the protein level via western blot analysis (Figure 5D and E). However, exogenous expression of BIRC5 did not induce changes in LAG3 mRNA level (data not shown). By recruiting mTOR inhibitor-Rapamycin, we blocked mTOR phosphorylation and partially reversed the PD-L1 overexpression caused by Lv-BIRC5 (Figure 5F and G). These results indicated that BIRC5 promotes PD-L1 expression in NSCLC cells via mTOR activation.

### BIRC5 promotes tumor growth and PD-L1 expression *in vivo*

**Figure 6:**
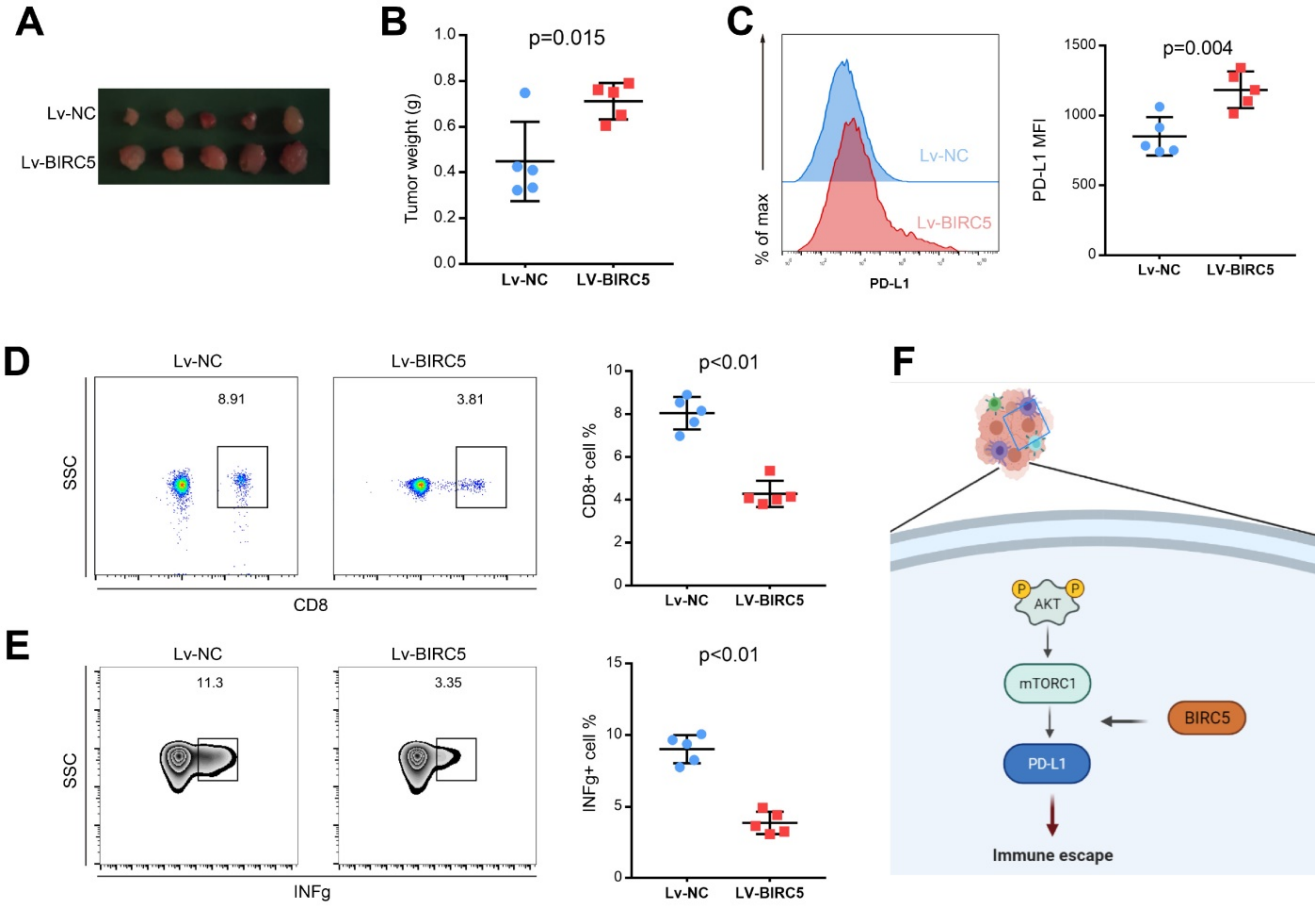
Overexpression of BIRC5 enhanced lung cancer progression *in vivo*. **(A and B)** BIRC5 overexpression markedly increased the tumor burden. **(C)** Flow cytometry analysis detected the mean fluorescence intensity (MFI) of PD-L1 was increased in the Lv-BIRC5 group compared to that in the control group. **(D)** The infiltration of CD8 positive T cells was significantly decreased in the Lv-BIRC5 group compared with the controls. **(E)** CD8 positive T cells in the Lv-BIRC5 group have a lower potential to secrete IFN-g. **(F)** The illustrated mechanism of BIRC5 participating in immune escape of lung adenocarcinoma.

To determine the function of overexpressed BIRC5 on tumor growth *in vivo*, a tumor model using the LLC cell line was recruited. LLC cells transfected with Lv-NC or Lv-BIRC5 were injected into C57BL/6 mice. Assessments of tumor weight revealed that ectopic BIRC5 markedly enhanced tumor growth (Figures 6A and B). In addition, we investigated how BIRC5 affected PD-L1 expression in lung cancer cells *in vivo*. According to flow cytometry analysis, the mean fluorescence intensity (MFI) of PD-L1 decreased significantly in the Lv-BIRC5 group rather than the Lv-NC group, as shown in Figure 6C. Since the PD-1/PD-L1 signaling pathway axis modulates an allosteric immune response, we wondered if BIRC5 overexpression in lung cancer cells would upregulate PD-L1 and encourage immune escape. The ratio of tumor-infiltrating CD8 T cells (TILs) in the Lv-BIRC5 group was significantly lower than that in the control group (Figure 6D), just as was the proportion of functional IFN-g secreted by TILs (Figure 6E).

## Discussion

As one of the most properly studied human IAPs genes, BIRC5 is supposed to disappear in differentiated mature cells (except for a few specific cell types) 19, 20. It is reported that BIRC5 is the most frequently amplified gene on chromosome 17 with an amplification rate of 32.9% in lung cancer samples 21. In the last decade, a clinical study of YM155 (a BIRC5 suppressor) with carboplatin and paclitaxel in advanced NSCLC patients was carried out, however, ended up with no improvement in the response rate of the chemotherapy 22. In a recent study on hepatocellular cancer, BIRC5 was found to be one of the top scorers with the best prognostic value that high expression of BIRC5 significantly correlated to weaker immune cells infiltrations and poorer overall survival 23. As immunotherapy is becoming one of the major strategies for LUAD, especially for advanced ones 24, BIRC5's prognosis value and its correlation with TME are supposed to be of great significance. The present study is the first one that recruited the TCGA-LUAD cohort to research the biological roles of BIRC5, aiming to validate BIRC5 can serve as not only a significant biomarker for LUAD diagnosis and prognosis but also an indicator for LUAD immunotherapy.

First of all, we discovered that BIRC5 expression was significantly higher in LUAD samples than in normal and adjacent ones. According to KM-plotter analysis, patients with LUAD had shorter OS, FP, and PPS (P<0.001). Multivariate Cox analysis indicated that aberrant BIRC5 expression was an independent risk for the prognosis of LUAD patients. Furthermore, high BIRC5 expression was associated with clinicopathologic features such as T classification, N stage, and pathologic stage. The results showed that high BIRC5 expression was associated with advanced stages, implying that it could be used as a marker to distinguish between early and advanced LUAD. What's more, the ROC analysis confirmed the diagnostic value, and a prognostic nomogram using age, gender, T stage, N stage, M stage, pathologic stage, and BIRC5 was created, indicating that BIRC5 expression could help with identifying high-risk LUAD patients.

Trafficking of immunogenic T cells to the TME is crucial for the successful immune response against malignancies, and infiltration of tumor-reactive T cells is related to improved cancer patient survival including LUAD 25-27. T cells with CD4 positive are considered to be the majority of total T lymphocytes, and they are generally classified as Th1 or Th2 based on the cytokines secreted. Th1 cells mainly produce cytokines that defend against the tumor, such as TNF-a and IFN-γ, while Th2 cells promote tumor growth by releasing cytokines that inhibit the antitumor immune responses 28. Though several studies have claimed BIRC5 was related to immune signature in several solid tumors, few of them researched the detailed mechanisms 23, 29-31. In this study, we analyzed the data from the TCGA-LUAD cohort and found the high BIRC5 level was associated with chemokine activity, cytokine activity, and pathways in immune cells chemotaxis, which is deeply involved in TME of LUAD 32-34.

Increasing tumor antigen-reactive T cell infiltration is a promising strategy for immunotherapy for cancers. Recently, Huang et al. reported that targeting the RGS-1 in tumor-specific T cells improves their immigration to breast cancer and further enhances the response to checkpoint inhibition of PD-L1 35. Another study conducted by Liu et al. found antagonist of ADORA1 promotes tumor PD-L1 expression via the cAMP-CREB-ATF3 axis and improves immunotherapy efficacy by increasing CD8 positive T cell activity in lung cancer 36. Given most LUAD with TPS<1% can hardly benefit from immunotherapy targeting PD-L1, studies discovering more targets and corresponding adjuvants are in urgent need. We detected significant correlations between BIRC5 expression and levels of classic genes representing exhausted T cells, including PD-1, PD-L1, LAG3, and TIM-3. Furthermore, we discovered a decrease in the number of CD8 T cells in tumors that grew from LLC cells with BIRC5 overexpression, which was accompanied by a depletion of IFN-g levels. It is the first study applies *in vivo* experiments to research the function of BIRC 5 on LUAD immunity, which proved a significant CD8 T cell exhaustion following the increase of BIRC5.

Our findings imply that BIRC5 has great value in diagnosis and predicting survival. More importantly, BIRC5 promotes lung cancer progression partially by modulating PD-L1 expression via mTOR signaling and inducing tumor immune evasion. Though clinical trials of BIRC5 combined chemotherapy failed to improve the efficacy of treatment for LUAD 22, its potential application in combination with checkpoint inhibitors could be a new insight into LUAD therapy.

## Supplementary Material

Supplementary figure.

## Figures and Tables

**Table 1:** The relationship between BIRC5 levels and clinical features

Characteristic	Low BIRC5 (n=256)	High BIRC5 (n=257)	p value
**T stage (n=510)**	n=255	n=255	0.002
T1	104 (20.4%)	64 (12.5%)	
T2	118 (23.1%)	158 (31%)	
T3	23 (4.5%)	24 (4.7%)	
T4	10 (2%)	9 (1.8%)	
**N stage (n=501)**	n=248	n=253	0.004
N0	181 (36.1%)	149 (29.7%)	
N1	39 (7.8%)	56 (11.2%)	
N2	28 (5.6%)	46 (9.2%)	
N3	0 (0%)	2 (0.4%)	
**M stage (n=369)**	n=175	n=194	0.328
M0	166 (45%)	178 (48.2%)	
M1	9 (2.4%)	16 (4.3%)	
**Pathologic stage (n=505)**	n=251	n=254	0.002
Stage I	157 (31.1%)	117 (23.2%)	
Stage II	52 (10.3%)	69 (13.7%)	
Stage III	32 (6.3%)	52 (10.3%)	
Stage IV	10 (2%)	16 (3.2%)	
**Gender (n=513)**	n=256	n=257	< 0.001
Female	159 (31%)	117 (22.8%)	
Male	97 (18.9%)	140 (27.3%)	
**Age (n=494)**	n=250	n=244	0.073
≤65	110 (22.3%)	128 (25.9%)	
>65	140 (28.3%)	116 (23.5%)	
**Smoker (n=499)**	n=249	n=250	0.056
No	45 (9%)	29 (5.8%)	
Yes	204 (40.9%)	221 (44.3%)	
**OS event (n=513)**	n=256	n=257	< 0.001
Alive	183 (35.7%)	143 (27.9%)	
Dead	73 (14.2%)	114 (22.2%)	

NOTE: Some of the LUAD samples were included in the TCGA with incomplete clinical information, which leads to inconsistencies in the case numbers in the clinic parameters and the subhead (e.g. Three LUAD patients included in this study had no information on the T stage in the TCGA database. So, only 510 cases, three less than the total number 513, were included in the analysis of the T stage).

**Table 2:** BIRC5 expression was associated with poor prognostic clinical characteristics in patients with LUAD

Characteristics	Total (N)	Odds Ratio (OR)	P value
Age (>65 vs. ≤65)	494	0.712 (0.499-1.014)	0.060
Gender (Male vs. Female)	513	1.961 (1.381-2.795)	<0.001
Smoker (Yes vs. No)	499	1.681 (1.021-2.807)	0.043
T stage (T2&T3&T4 vs. T1)	510	2.055 (1.413-3.008)	<0.001
N stage (N1&N2&N3 vs. N0)	501	1.886 (1.298-2.754)	<0.001
M stage (M1 vs. M0)	369	1.658 (0.727-4.014)	0.240
Pathologic stage (Stage III&Stage IV vs. Stage I&Stage II)	505	1.819 (1.185-2.819)	0.007
Primary therapy outcome (PD&SD vs. PR&CR)	426	1.600 (1.028-2.509)	0.039
Residual tumor (R1&R2 vs. R0)	361	1.287 (0.483-3.617)	0.618
Anatomic neoplasm subdivision (Right vs. Left)	498	1.066 (0.744-1.526)	0.728
Peripheral Lung vs. Central Lung	189	1.011 (0.547-1.861)	0.971
Number_pack_years_smoked (≥40 vs. <40)	351	1.457 (0.958-2.223)	0.080

NOTE: Some of the LUAD samples were included in the TCGA with incomplete clinical information, which leads to inconsistencies in the case numbers in the clinic parameters and the subhead. (e.g. 19 LUAD patients included in this study had no information of their age in the TCGA database. So, only 494 cases, 19 less than the total number of 513, were included in the analysis of the age).
