# Supplementary material for: BIRC5 Modulates PD-L1 Expression and Immune Infiltration in Lung Adenocarcinoma
Source: J Cancer. 2022 Aug 21;13(10):3140–50. doi: 10.7150/jca.69236 (PMC9414029; doi:10.7150/jca.69236)

Supplementary Figure 1 Cox analysis of BIRC5 in TCGA-LUAD cohort. (**A)** Forest plot of univariate Cox proportional hazards regression analysis of the Overall Survival (OS) in TCGA-LUAD cohort. **(B)** Forest plot of univariate Cox proportional hazards regression analysis of Progress Free Survival (PFS) in TCGA-LUAD cohort. (**C)** nomogram for predicting probability of patients with 1-, 3- and 5-year OS.

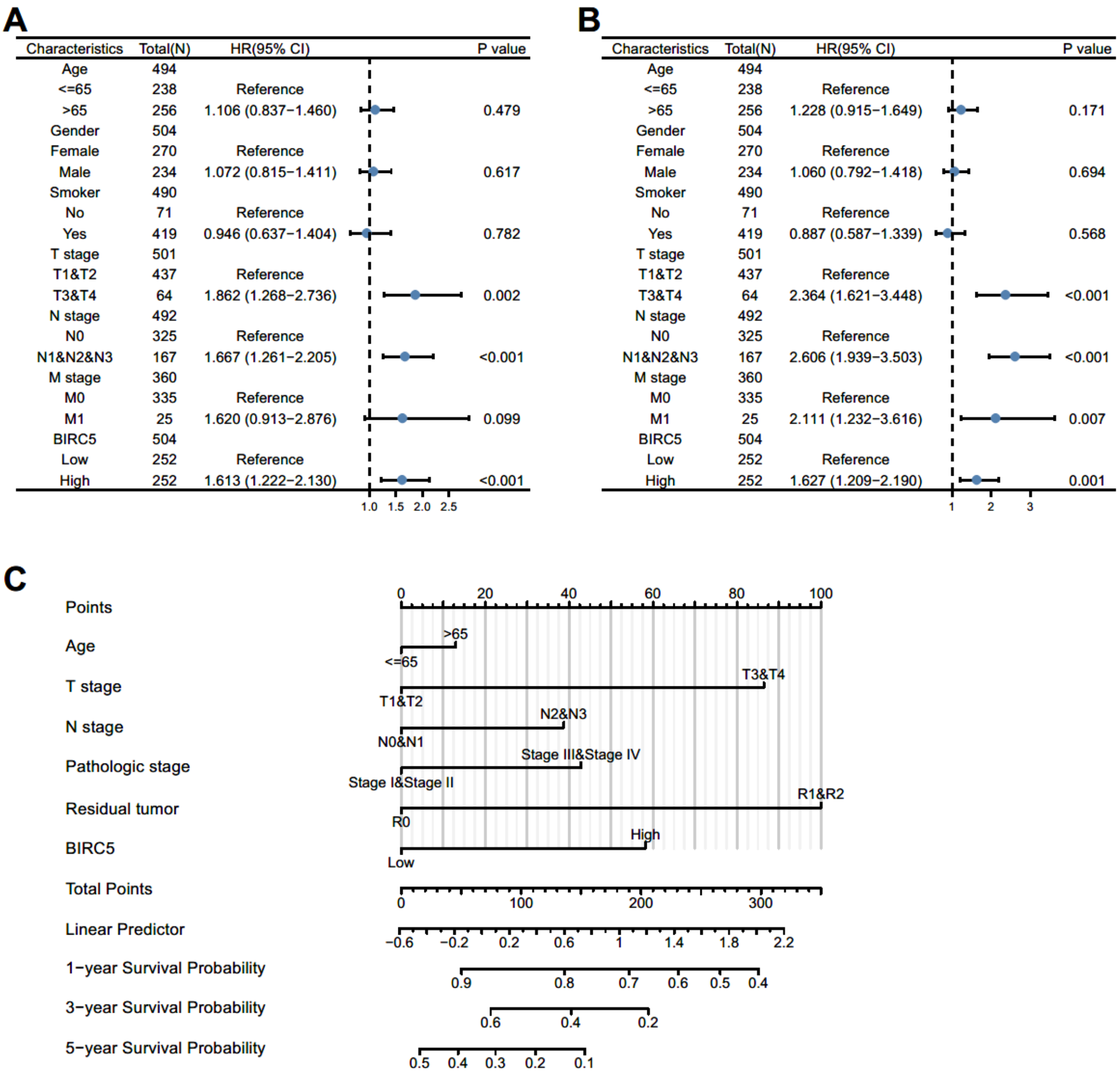

Supplement: Supplementary file 1 — Supplementary figure. [file jcav13p3140s1.pdf]
